# Impaired Activity of Blood Coagulant Factor XIII in Patients with Necrotizing Enterocolitis

**DOI:** 10.1038/srep13119

**Published:** 2015-08-17

**Authors:** Guo-Zhong Tao, Bo Liu, Rong Zhang, Gigi Liu, Fizan Abdullah, Mary Cay Harris, Mary L. Brandt, Richard A. Ehrenkranz, Corinna Bowers, Camilia R. Martin, R. Lawrence Moss, Karl G. Sylvester

**Affiliations:** 1 Department of Surgery, Stanford University School of Medicine, Stanford, USA; 2 Department of Surgery, Johns Hopkins University School of Medicine, Baltimore, USA; 3 Department of Pediatrics, Children’s Hospital of Philadelphia, Philadelphia, USA; 4 Department of Surgery, Texas Children’s Hospital, Baylor College of Medicine, Houston, USA; 5 Department of Pediatrics, Yale University School of Medicine, New Haven, USA; 6 Division of Pediatric Surgery, Nationwide Children’s Hospital, Columbus, USA; 7 Department of Surgery, Ohio State College of Medicine, Columbus, USA; 8 Department of Neonatology, Beth Israel Deaconess Medical Center, Boston, USA; 9 Lucile Packard Children’s Hospital Stanford, Stanford, USA; 10 Center for Fetal and Maternal Health, Stanford Children’s Health, Stanford, USA

## Abstract

Necrotizing enterocolitis (NEC) is the most common gastrointestinal (GI) medical/surgical emergency of the newborn and a leading cause of preterm neonate morbidity and mortality. NEC is a challenge to diagnose since it often shares similar clinical features with neonatal sepsis. In the present study, plasma protein profiling was compared among NEC, sepsis and control cohorts using gel electrophoresis, immunoblot and mass spectrometry. We observed significant impairment in the formation of fibrinogen-γ dimers (FGG-dimer) in the plasma of newborns with NEC that could efficiently differentiate NEC and sepsis with a high level of sensitivity and specificity. Interestingly, the impaired FGG-dimer formation could be restored in NEC plasma by the addition of exogenous active factor XIII (FXIII). Enzymatic activity of FXIII was determined to be significantly lower in NEC subject plasma for crosslinking FGG when compared to sepsis. These findings demonstrate a potential novel biomarker and related biologic mechanism for diagnosing NEC, as well as suggest a possible therapeutic strategy.

Necrotizing enterocolitis (NEC) is a devastating acquired intestinal disease that afflicts up to 10% of preterm infants of extremely low birth weight (ELBW)^1^. Late diagnosis and delays in treatment of NEC can result in progressive cases with devastating consequences including loss of significant intestinal length, prolonged critical illness and neurodevelopmental impairment^2^. Despite decades of research no disease marker has emerged with clinically sufficient sensitivity and specificity. Most reported biomarkers of NEC (CRP, IL-6, IL-10 and SAA etc.) are responsive to systemic inflammation and therefore have relatively high sensitivities, but lack disease specificity to efficiently differentiate NEC from neonatal sepsis^3^,^4^. In contrast, biomarkers associated with intestinal damage such as I-FABP or claudin-3 possess greater specificity, but are limited by diminished sensitivity and detect NEC more reliably after disease progression^3^,^4^,^5^,^6^. Since NEC shares similar clinical signs and is often confused with neonatal sepsis, especially when it has concurrent bloodstream infection^7^, a reliable biomarker for differentiating the two conditions is urgently needed.

The tissue response to injury is initiated by activation of the coagulation cascade to prevent blood loss and initiate wound healing. FXIIIa, a transglutaminase of the coagulation cascade, covalently crosslinks two molecules of fibrinogen-γ (FGG-dimer) to serve a key role during the final stage of fibrin clot formation. During wound healing, particularly in the gastrointestinal tract (GI) tract constantly faced with digestive enzymes and peristaltic movement, it is imperative to form a stable fibrin clot on the surface of injured tissue to avoid proteolytic or mechanical disruption. Without FXIII activity, a fibrin clot forms normally but prematurely breaks down, resulting in recurrent bleeding and an overall delay in the process of wound healing^8^. In the present study, we discovered that FGG-dimers were absent from the plasma of NEC patients, and that this observation efficiently distinguished NEC from sepsis. Further investigation of the molecular mechanism revealed significantly impaired FXIII activity in the plasma of subjects with NEC and implied a possible therapeutic strategy for this disease.

## Results and Discussion

### Disappearance of FGG-dimer is a potential biomarker for differentiating NEC from neonatal sepsis

**Figure 1:**
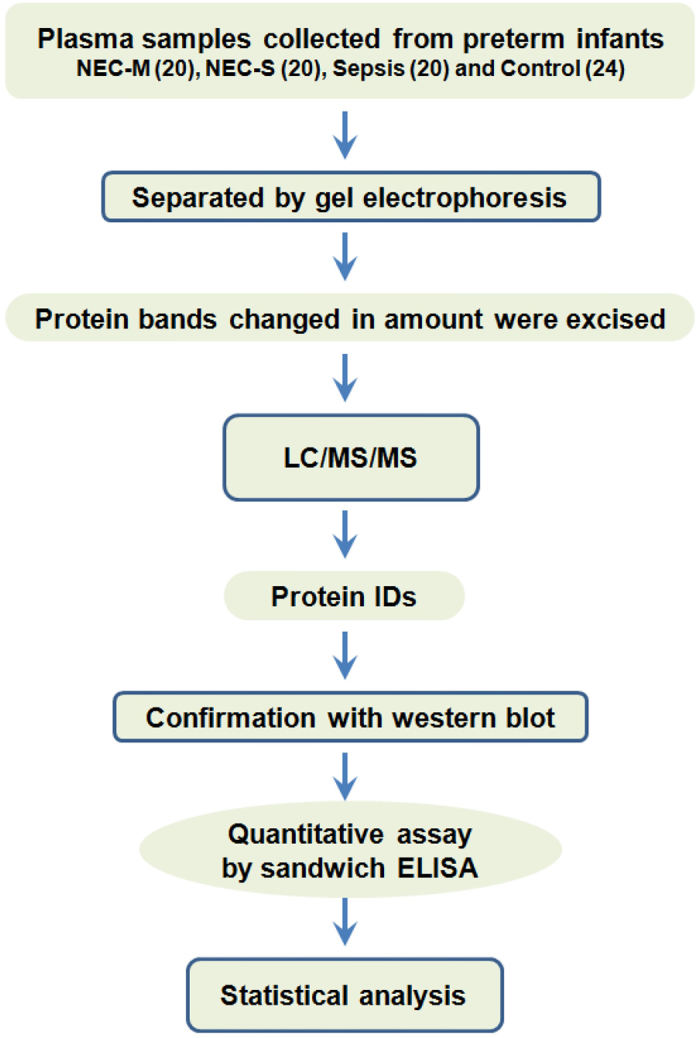
Study design for discovery of potential plasma biomarkers to distinguish NEC and neonatal sepsis.

**Figure 2:**
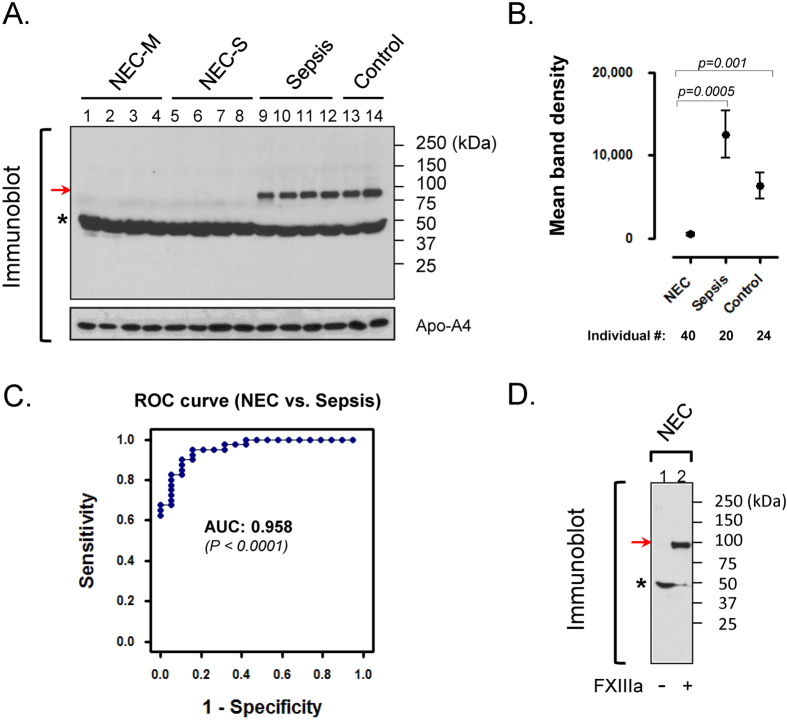
Lack of crosslinked FGG-dimers in plasma can efficiently differentiates NEC from neonatal sepsis. (**A**) Crosslinked FGG species are absent from the plasma of infants with NEC. Equal amounts of pooled plasma (n = 5) from indicated groups were separated on SDS-PAGE, followed by immunoblot using a monoclonal antibody to FGG. Notably, FGG-monomers (indicated by asterisk) were detected in all four groups (lanes 1–14); in contrast, a FGG-dimer species (~100 kDa, indicated by arrow) was detectable only in sepsis (lanes 9–12) and control (lanes 13, 14) but not in NEC-M/S (lanes 1–8) plasmas. (**B**) FGG-dimer formation was individually analyzed in each sample by immunoblot using FGG antibody followed by density analysis of the identified protein bands using ImageJ software. (**C**) Receiver-operating characteristic (ROC) curve analysis for assessment of potential clinical utility. The data from panel-2B was statistically analyzed by ROC curve and demonstrated superior sensitivity and specificity (AUC = 0.958). (**D**) Addition of exogenous FXIIIa *in vitro* restored FGG-dimer formation in NEC samples. Pooled NEC (M/S) plasmas were incubated with (lane-2) or without (lane-1) exogenous active FXIII for 10 min. Dimer-formation was assessed by immunoblot using FGG-antibody. Note that the exogenous FXIII-induced reconstitution of FGG-dimers suggests a likely impairment of FXIII activity in NEC.

The study procedures are outlined in Fig. 1. During the characterization of plasma samples, a protein band (52 kDa) was observed to increase in NEC compared to control or sepsis infants as indicated by the arrows (suppl Fig. 1). The altered protein band was analyzed using mass spectrometry and identified as likely FGG (suppl Fig. 2A,B). For further verification, the samples were resolved by SDS-PAGE followed by immunoblot using two independent monoclonal antibodies to FGG. Both antibodies recognized the expected protein band migrating at 52 kDa (FGG-monomer); however, an additional protein species (~100 kDa) was also detected in sepsis (lane 9–12) and control (lane 13, 14) but surprisingly not in plasma from early and late NEC (lane 1–8) of Fig. 2A. The larger protein species (FGG-dimer) appeared to be related to FGG-monomer, but due to its striking alteration, it is superior to the quantitative changes in FGG-monomer as a distinguishing feature. Upon examining individual samples by immunoblot, the ~100 kDa FGG-dimer band was notably detectable in all sepsis (20/20; 100%) cases with high average band density 11746.4 ± 2728.5 and 22 of 24 controls (6612.5 ± 1581.0), but a mere 15 of 40 in NEC-M/S (with very low density 524.1 ± 225.3) plasmas (Fig. 2B). These results yielded highly significant class distinction between NEC and Sepsis (AUC = 0.958; *p* < *0.0001*) (Fig. 2C) as well as NEC and control (AUC = 0.91; *p* < *0.0001*) (Supplemental Fig. 3). ROC curve analysis, a statistical tool for evaluating the diagnostic power of a biomarker, indicated that the FGG-dimer detection has excellent overall accuracy with high sensitivity and specificity (Fig. 2C).

### Restoration of FGG-dimer formation in NEC by addition of exogenous FXIIIa

Next, we sought to determine the mechanism explaining the absent FGG-dimer species that is accompanied by a minor increase in FGG-monomer in the plasma of infants with NEC (Fig. 2A). Previous studies have characterized FGG-dimer cross-linking *in vitro* in response to active factor XIII^9^,^10^. Additionally, elevated FGG-dimer has been identified in the plasma of cancer patients^11^ as well as in mouse liver after Fas-induced apoptosis^12^. Accordingly, as shown in Fig. 2D, the addition of exogenous active (a) FXIII restored the ability to crosslink FGG in NEC plasma. In addition, we determined that the observed FGG-dimer differed from the D-dimer assay (Supplemental Fig. 4) currently in widespread clinical use for detecting fibrin degradation products that elevate in blood when disseminated intravascular coagulation (DIC) occurs. A reduced platelet count observed in surgical but not early stage NEC (Supplemental Table 1) is consistent with advanced tissue necrosis but does not itself indicate DIC in progressive disease. Since the absence of FGG-dimer was detected at both early and late NEC (Fig. 2A), the alteration is unlikely a consequence of DIC. In addition, there was no evidence to indicate that total fibrinogen was consumed and therefore decreased, especially in early NEC, *i.e.* no difference in Fibrinogen between early and late NEC (Fig. 2. lanes 1–4 vs. lanes 5–8 or 9–14).

Taken together, these data indicate that the 100kDa-protein species is a dimer of FGG covalently crosslinked by FXIIIa. Based on the undetectable level of cross-linked FGG-dimer in NEC plasma, we speculate that infants with NEC may possess impaired FXIII activity. The significance of a relative FXIII deficiency could include a diminished ability of the gut to recover from mucosal injury^13^.

### Impaired activity of plasma FXIII found in NEC patients

**Figure 3:**
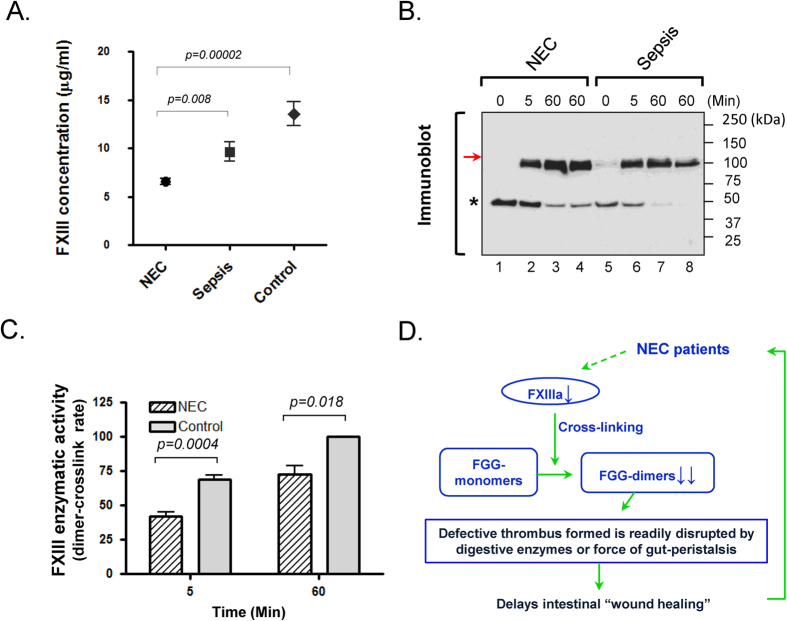
Absence of cross-linked FGG-dimers is correlated with insufficient enzymatic activity of FXIII in the plasma of NEC patients. (**A**) Reduced plasma level of FXIII in NEC patients. Quantitative measurement was performed among the indicated groups using sandwich ELISA. Plasma levels of FXIII in NEC is significantly reduced when compared to control or sepsis cohorts. (**B**) Crosslinking activity of endogenous FXIII was assessed *in vitro* by FGG*-*dimer formation after the addition of FXIII activators. Pooled NEC and sepsis plasmas were incubated with the same amount of CaCl_2_ and thrombin (known FXIII activator) at 25 °C (lanes 1–3, 5–7) or 37 °C (lanes 4, 8) for the indicated time points. Dimer formation was detected by immunoblot using FGG-antibody. A representative result is shown from three independent experiments. (**C**) Reduced enzymatic activity of FXIII in NEC plasma. The protein band densities of immunoblot of panel-C were scanned, analyzed using NIH imageJ software and graphed for relative comparisons. Note that the rates of newly formed dimer vs. total (dimer + monomer) FGG bands are significantly lower in NEC compared to sepsis samples at 5 or 60 min, indicating that plasma from NEC subjects has lower enzymatic activity of FXIII than sepsis. (**D**) Schematic working model for the impact of reduced FXIII activity on intestinal “wound healing” in NEC patients. FXIIIa functions as a transglutaminase to covalently crosslink two molecules of FGG at the last step of the blood coagulant cascade. Without this step thrombus forms on “wounds” but is unstable, readily breaking down especially in the GI-tract given the constant exposure to both digestive enzymes and peristaltic movement. It is conceivable that this consequently delays the healing process of intestinal injuries in GI-diseases like NEC.

As demonstrated by Fig. 3A, FXIII protein concentration was significantly lower in NEC (6.56 ± 2.21 μg/ml) compared to control (13.55 ± 5.38 μg/ml) (p = 0.00002) or the sepsis cohort (9.63 ± 4.49 μg/ml) (p = 0.008), indicating an inverse relationship between plasma FXIII levels and NEC. Additionally, upon further stratified analysis for subsets of NEC, no significant difference (p = 0.1893) was found between NEC-M (7.03 ± 1.97 μg/ml) and NEC-S (6.13 ± 2.37 μg/ml) cohorts though it appeared to be negatively correlated between FXIII plasma level and severity of the disease. We further sought to determine the enzymatic activity of FXIII since recently reported genomic mutations^14^ can yield impaired FXIII function despite measurable plasma levels. FXIII transglutaminase activity was assessed by the *in vitro* ability to cross-link FGG-monomers to dimers (Fig. 3B) and expressed as the crosslinking rate [(newly formed FGG-dimer)/total FGG (dimer + monomer)] × 100%. In comparison to sepsis, total FXIII activity was significantly decreased in NEC plasma at the indicated reaction time points (p = 0.004 and 0.018, respectively) as shown in Fig. 3B,C. These data reveal that the observed quantitative and total activity deficiency in FXIII are closely associated with the molecular mechanism accounting for absent FGG-dimers, and further suggest that replenishment of FXIII may be a potential therapeutic strategy for facilitating intestinal “wound healing” in NEC patients based on the current working model (Fig. 3D). Due to the unique nature of GI-tract injuries that include constant exposure to a variety of digestive enzymes and intestinal peristalsis, stable clot formation on the epithelial surface may be required more for tissue recovery and maintenance than in other tissues like skin and muscle. Although it is currently unknown whether the observed impaired FXIII activity is a causative factor for NEC, we speculate that intestinal “wound healing” may be accelerated or preserved by the administration of active FXIII as part of a novel treatment or prevention strategy.

## Patients and Methods

### Study subjects

Human plasma samples were obtained from preterm neonates with NEC (n = 40; 20 medical and 20 surgical), sepsis (n = 20) and healthy controls (n = 24) through a US multi-institutional study consortium that included Stanford University, Johns Hopkins University, Children’s Hospital of Philadelphia, Baylor College of Medicine, Yale University, Nationwide Children’s Hospital, Ohio State College of Medicine, Beth Israel Deaconess Medical Center and Lucile Packard Children’s Hospital Stanford. All plasma samples were obtained from infants treated at one of the collaborating institutions and were collected at the time of initial clinical concern for disease (NEC or sepsis) – a point at which definitive diagnosis was not able to be determined on clinical grounds alone. Patients with a previous diagnosis of NEC or sepsis, a history of prior abdominal surgery, or a known congenital anomaly of the gastrointestinal tract or abdominal wall were excluded from the study. Infants were followed clinically and ultimately categorized as either medical NEC (improved without surgery) or surgical NEC (required laparotomy, peritoneal drainage, or died from complications of NEC prior to intervention). Patient inclusion was ultimately confirmed by the presence of signs specific for NEC by Bell’s criteria (pneumatosis intestinalis) or, for the sepsis group, by either positive blood cultures or a clinical syndrome associated with a high probability of infection. All subjects were assigned a modified Bells Stage for NEC (supplemental Table 1) according to previously published methods^15^ and as demonstrated in Supplemental Figure 5. The blood culture results for NEC and sepsis are listed in supplemental Table 2. Control subjects were identified as premature infants in the NICU without known or suspected inflammatory disease. All subjects above were selected based on a similar mean birth weight and a mean gestational age (all ≤33 weeks) as shown in Table 1. 100 μl of blood was centrifuged and plasma was stored at –80°C until batch interrogation. This study was approved by the human subjects research committee at each institution named above. The methods utilized were carried out in accordance with approved guidelines and regulations. Written informed consent was obtained from the parents of all subjects for blood sampling.

### Screening of potential biomarkers and Assessment of FGG-dimer formation

For the discovery of candidate markers of NEC, plasma was analyzed with 12% SDS-PAGE and protein bands that were visually altered between NEC and control groups were individually excised from the gels for further mass spectrometry. Identification (ID) of proteins were obtained by collecting LC-MS/MS data, then searching the MS/MS spectra against existing databases using scaffold-4 software at Stanford University Mass Spectrometry facility (Stanford, CA). FGG-monomer and -dimer were assessed by their different migration on SDS-PAGE (52 and 100 kDa, respectively).

### *In vitro* cross-linking of FGG and quantitative FXIII assay

For restoration of FGG-dimer formation in NEC plasmas, exogenous FXIIIa was added to pooled NEC plasmas, and incubated at 25 °C for 10 min. For assessment of endogenous FXIII activity, pooled plasmas were incubated with same amount of CaCl_2_ and thrombin (a known FXIII activator) in HEPES buffer at 25 °C or 37 °C for the indicated time points. The covalently cross-linked dimers were determined by immunoblot using FGG-antibody. Human FXIII ELISA kit (ab108836) was used for quantification according to the manufacturer’s instructions.

### Statistics

Statistical significance between different groups was assessed using GraphPad software’s chi-square test, one-way ANOVA and student’s t-test. *P* value of less than 0.05 was considered to be significant.

## Additional Information

**How to cite this article**: Tao, G.-Z. *et al.* Impaired Activity of Blood Coagulant Factor XIII in Patients with Necrotizing Enterocolitis. *Sci. Rep.* **5**, 13119; doi: 10.1038/srep13119 (2015).

## Supplementary Material

Supplementary Information

## Figures and Tables

**Table 1:** Demographic information of human preterm infants chosen by this study.

	**NEC**	**Sepsis**	**Control**	**p-value**
	**N = 40**	**N = 20**	**N = 24**	
Gender				
Male	25 (62.5%)	9 (47.4%)	12 (50%)	0.451^b^
Female	15 (37.5%)	10 (52.6%)	12 (50%)	
Race				
Asian	6 (15%)	3 (15.8%)	3 (12.5%)	0.091^b^
Black	13 (32.5%)	1 (5.3%)	9 (37.5%)	
Native Hawaiian or Pacific	0 (0%)	1 (5.3%)	0 (0%)	
White	20 (50%)	10 (52.6%)	10 (41.7%)	
Other	1 (2.5)	4 (21%)	2 (8.3%)	
Gestational Age^a^ (weeks)	27.7 ± 3.3	27.7 ± 2.7	28.3 ± 2.9	0.736^c^
Birth Weight^a^ (grams)	1034.6 ± 448.6	1052.9 ± 441.0	1093.9 ± 473.9	0.880^c^

(^a^Data is shown by mean ± SD; p-value is calculated by Chi-square^b^ or one-way ANOVA^c^ test).
