# Supplementary material for: Impaired Activity of Blood Coagulant Factor XIII in Patients with Necrotizing Enterocolitis
Source: Sci Rep. 2015 Aug 17;5:13119. doi: 10.1038/srep13119 (PMC4642514; doi:10.1038/srep13119)
Supplement: Supplementary Information [file srep13119-s1.pdf]

Supplemental data for:

Impaired Activity of Blood Coagulant Factor XIII in Patients with Necrotizing Enterocolitis

Guo-Zhong Tao[1], Bo Liu[1], Rong Zhang[1], Gigi Liu[1,2], Fizan Abdullah[2], Mary Cay Harris[3], Mary Brandt[4], Richard Ehrenkranz[5], Corinna Bowers[6,7], Camilia R. Martin[8], R. Lawrence Moss[6,7] and Karl G. Sylvester[1,9, 10]

[1]Department of Surgery, Stanford University School of Medicine, Stanford, USA

[2]Department of Surgery, Johns Hopkins University School of Medicine, Baltimore, USA

[3]Department of Pediatrics, Children's Hospital of Philadelphia, Philadelphia, USA

[4]Department of Surgery, Texas Children's Hospital, Baylor College of Medicine, Houston, USA

[5]Department of Pediatrics, Yale University School of Medicine, New Haven, USA

[6]Division of Pediatric Surgery, Nationwide Children's Hospital, Columbus, USA

[7]Department of Surgery, Ohio State College of Medicine, Columbus, USA

[8]Department of Neonatology, Beth Israel Deaconess Medical Center, Boston, USA

[9]Lucile Packard Children’s Hospital Stanford, Stanford, USA

[10] Center for Fetal and Maternal Health, Stanford Children’s Health, Stanford, USA

Corresponding Author:

Karl G. Sylvester, M.D.

Department of Surgery, Division of Pediatric Surgery

Stanford University School of Medicine

Hagey Laboratory for Regenerative Medicine

257 Campus Drive

Stanford, CA 94305-5148

Email: sylvester@stanford.edu

Phone: (650) 804-0597

Fax: (650) 725-557

Supplemental Table-1. Bell's stage and platelet count for enrolled patients with NEC

	Modified Bell's staging						Platelet count (~ x1000 per µl)
	IA	IB	IIA	IIB	IIIA	IIIB	Mean ± SD
**NEC-M** (n=20)	3	6	7	4	0	0	307 ±142
**NEC-S** (n=20)	0	0	0	0	3	17	190 ±100*

(Student *t*-test, *p=0.04 compared to NEC-M cohort)

Supplemental Table-2. Blood culture result for preterm infants enrolled in this study

		**NEC**	**Sepsis**
		N=40	N=20
Culture result			
	Positive	3	4
	Negative	32	7
	Unknown**	5	9
Microorganism			
	Candida	1	0
	Enterococcus faecalis	1	0
	Staphylococcus aureus	0	1
	Staphylococcus epidermidis	1	3

(**including pending/uncertain data for NEC or clinical sepsis)

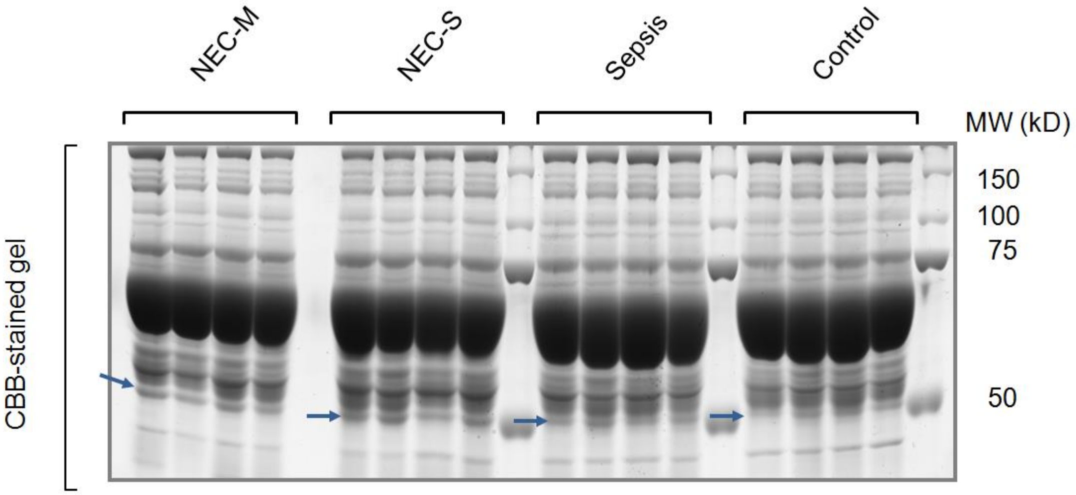

Supplemental ***Fig 1.*** *Comparison of plasma abundant proteins by SDS-PAGE*

Pooled plasma samples from indicated newborn cohorts were separated by one-dimensional SDS-PAGE and visualized by staining of coomassie brilliant blue. Note that protein bands indicated by arrows were altered among the groups and excised for MS/MS assay.

A.

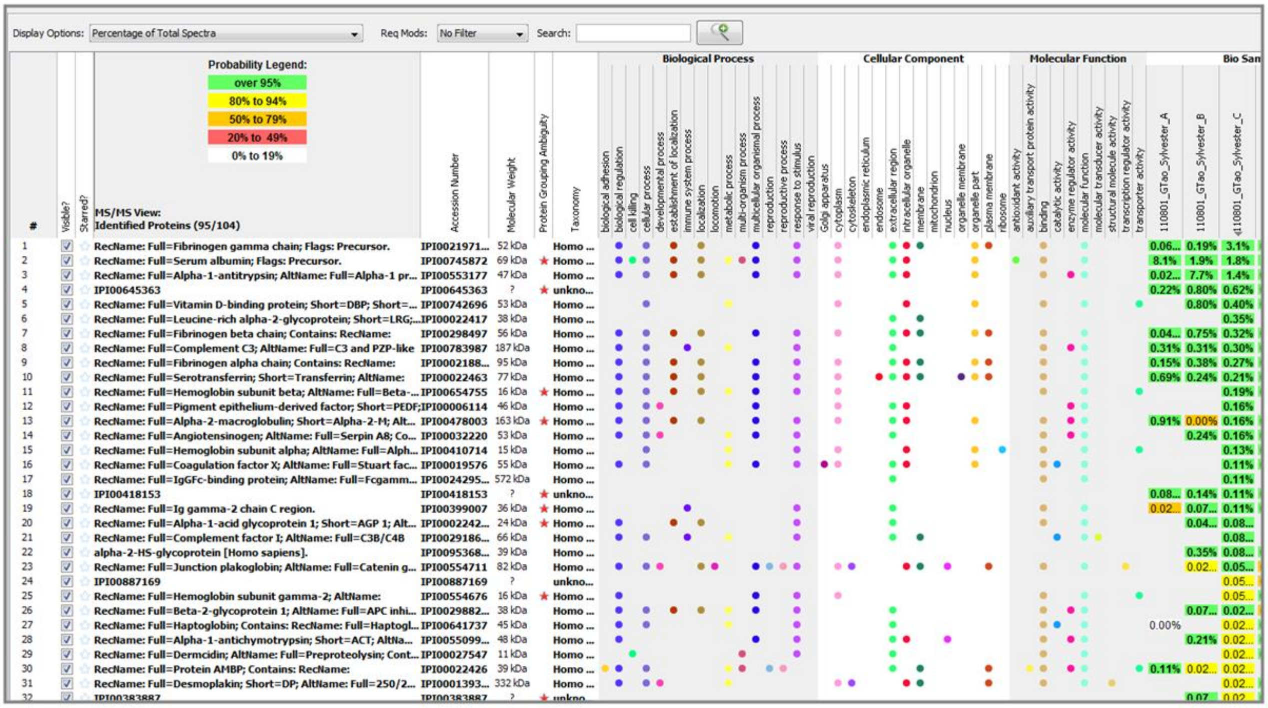

B.

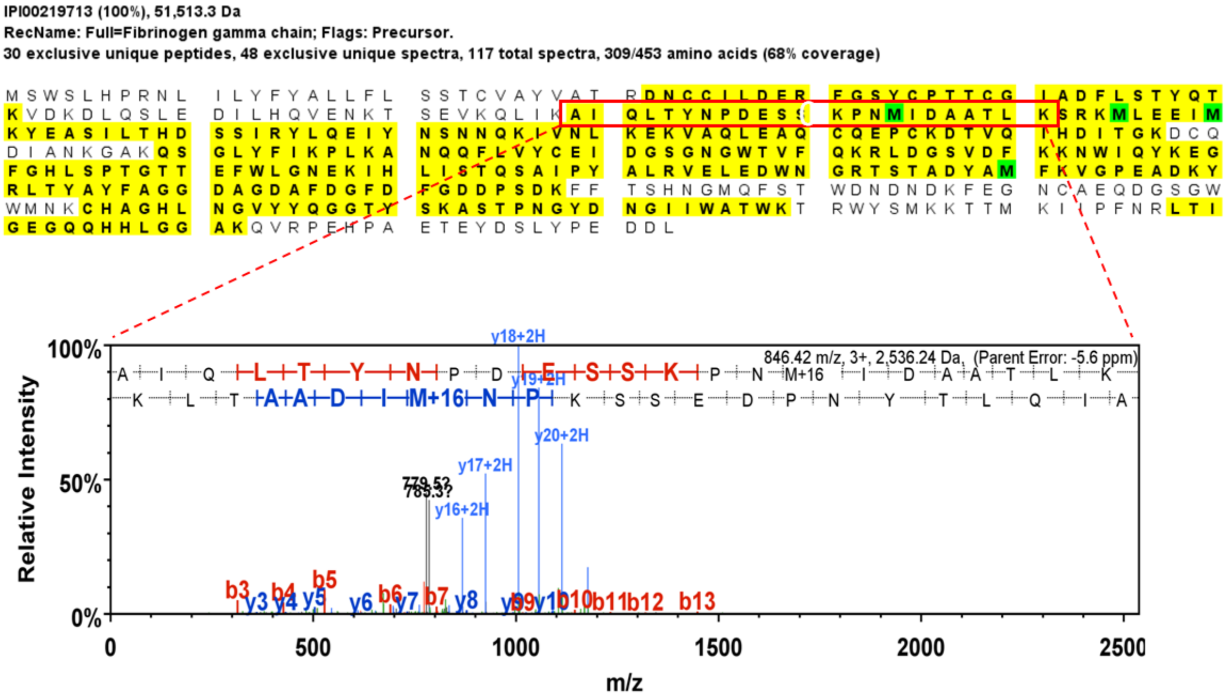

Supplemental ***Fig 2****. Identification of the target protein by mass spectrometry*

A) FGG as indicated by arrow was identified as the most likely candidate protein with the highest score. **B)** 68% of the full length FGG amino acid sequence (yellow highlight) was identified (upper), and spectra data from one peptide is shown as an example.

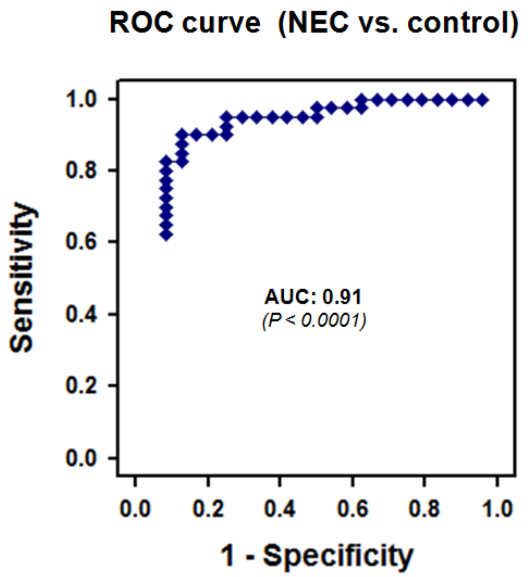

Supplemental ***Fig 3.*** *Receiver-operating characteristic (ROC) curve analysis for NEC and control neonates*

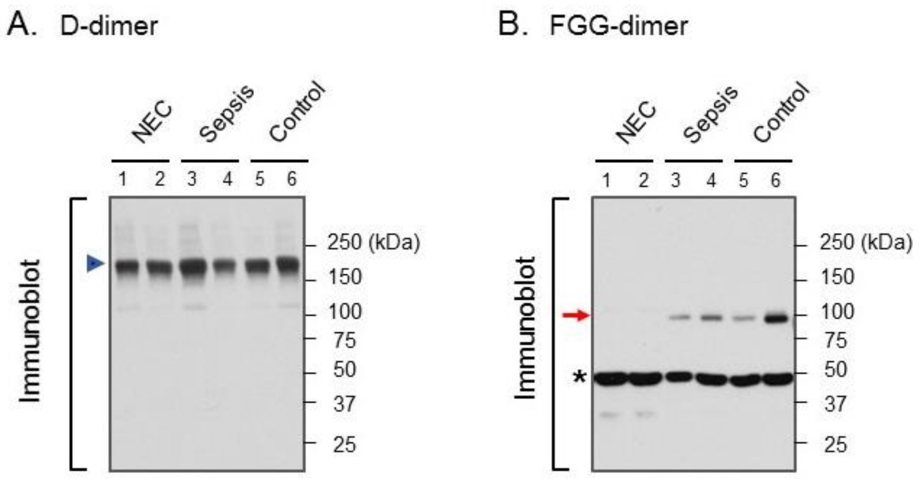

Supplemental ***Fig 4****. FGG-dimer detection is different from D-dimer assay*

Pooled plasma samples were subjected to immunoblot by D-dimer antibody after separation using non-reducing SDS-PAGE. A) D-dimer is detected at ~180kDa under non-reducing condition; but undetectable under reducing condition (not shown). Arrowhead is D-dimer protein band. B) The same plasma samples were analyzed by immunoblot using FGG monoclonal antibody and the ~100kDa protein band as indicated by the arrow is FGG-dimers. *Indicated FGG-monomer. Note that FGG-dimer differs from D-dimer.

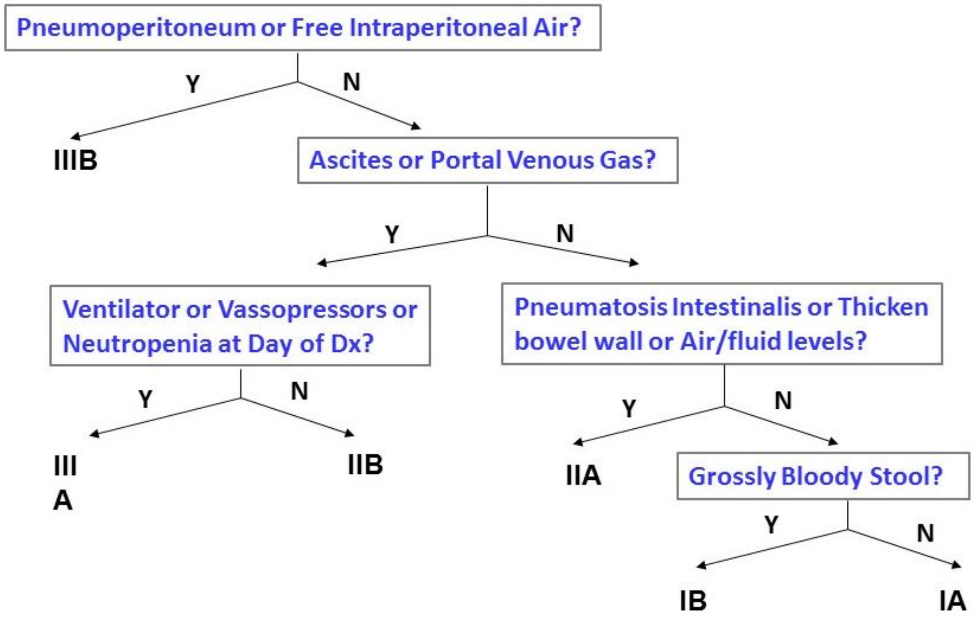

Supplemental ***Fig 5****. NEC classification by Bell's staging decision tree*
